# Experimental Study on the Manufacturing of Steel Inclined Walls by Directed Energy Deposition Based on Dimensional and 3D Surface Roughness Measurements

**DOI:** 10.3390/ma15144994

**Published:** 2022-07-18

**Authors:** Alejandro Pereira, Diego Carou, María Fenollera, Teresa Prado, Bartosz Gapiński, Michal Wieczorowski

**Affiliations:** 1 Faculty of Industrial Engineering, Campus Lagoas Marcosende, Universidade de Vigo, 36310 Vigo, Spain; mfenollera@uvigo.es; 2 Faculty of Aeronautical and Space Engineering, Universidade de Vigo, 32004 Ourense, Spain; 3 Faculty of Mechanical Engineering, Poznan University of Technology, Piotrowo Street 3, 60-965 Poznan, Poland; bartosz.gapinski@put.poznan.pl (B.G.); michal.wieczorowski@put.poznan.pl (M.W.)

**Keywords:** additive manufacturing, WAAM, GMAW, 3D topography, steel, inclined walls

## Abstract

Robotic-directed energy deposition has attracted the attention of the research community and industry as a process capable of producing large metallic parts. The selection of the manufacturing conditions is a critical step in improving the process efficiency and quality of the produced parts. The present work aims at analyzing the geometry and surface topography of walls built using several conditions and inclination angles, without additional supports except for the substrate. The walls were made of AWS A5.18. ER70S-6 steel using the Wire Arc Additive Manufacturing process. The study used both dimensional and 3D topography measurements to analyze the results. As findings, the travel speed played an important role in the size of the cross-section due to the heat input to the welding zone. Heat accumulation was a critical factor in the size and accuracy of the beads. Moreover, intermediate cooling provided structures with more uniform dimensions, smaller width, and higher layer growth. The inclination of the pieces influenced the width and uniformity of the beads, generating minor imperfections on the downside of the pieces because of gravity.

## 1. Introduction

Additive manufacturing has been in the spotlight in the last few years, becoming one of the key enabling technologies of the 4th Industrial Revolution [1]. Since the 1980s, additive manufacturing has evolved into a technology capable of producing high-quality parts in numerous materials, from polymers to metals [2]. The process offers the possibility to manufacture highly intricate structures that other processes cannot create [3].

The ISO 52900 standard [4] classifies additive manufacturing according to seven process categories. Among them, the Directed Energy Deposition (DED), Powder Bed Fusion (PBF), and sheet lamination processes allow manufacturers to produce metallic parts. In particular, the DED process offers advantages such as large part size, the possibility to work on even and uneven substrates, and a combination of materials [5].

Within the DED processes, additive manufacturing by wire arc (WAAM) allows the manufacture of metal parts in alloys based on Al, Ti, steel, Ni and bimetal, as in the case of CMT-based WAAM for NiTi shape memory alloys [6,7,8]. According to the ISO 17296-2 standard [9], in terms of filament-feed-based additive manufacturing methods, there are three families of heat sources: laser, welding arc and electron beam. The WAAM technology stands out for its high-energy utilization compared to its competitors: lasers (5%) and electron beams (20%) [10,11,12,13]. Moreover, it offers additional advantages such as the low cost of its equipment, no need to create a vacuum that is necessary for the electron beam-based processes, and it has a high deposition rate. In this sense, for steels, Selective Laser Melting (SLM) reaches deposition rates of 0.1 kg/h and Laser Metal Deposition (LMD) of 1 kg/h, while WAAM allows attaining up to 5–6 kg/h [14,15]. Several alternatives are available for controlling the motion during welding, such as conventional gantry systems and robotic-assisted welding. The latter is considered better diminishing the staircase effect, generally due to their available six degrees of freedom [16].

In the WAAM process, the accumulation of heat in successive layers is critical. Zhang et al. [17] investigated the heat transfer control system to evaluate the solder droplets’ frequency and size and, lastly, to improve precision. In this sense, applying the Cold Metal Transfer (CMT) technology is very appropriate to increase the wall production ratio by reducing the heat input. Chen et al. [18] have conducted experiments to study the stability of the formation of aluminum alloys using the WAAM-CMT system by using various process parameters and several industrial robot paths as well as Fronius digital welding modes. Yang et al. [19] have proposed using the Double Electrode–Gas Metal Arc Welding (DE-GMAW) process to minimize the energy transfer to the base metal, studying the characteristics of the forming process.

The main disadvantages of the WAAM process are the need to provide a substrate material on which the material is deposited, which finally will have to be removed, the lack of dimensional precision, and the poor quality of the surfaces of the generated parts [20]. Another important quality problem that can occur is the generation of porosities that decrease the mechanical properties of the parts [21,22]. For instance, during the welding process, harmful residual stresses may appear, modifying the geometry of the parts. Thus, the parts require machining to meet geometry and surface finish requirements. The use of welding conditions that minimize the energy input through CMT welding systems [23] can decrease the appearance of residual stresses.

As stated above, among the main advantages of the WAAM process is its high deposition rates. It should also be noted that the larger the robot’s size, the greater the ability to manufacture larger parts. Moreover, greater flexibility can be attained when increasing the degrees of freedom of the robot. For instance, a seven-axis robot consisting of a rotary table increases the possibility of using movable support independent of the welding torch and varying the inclination of the welding torch, even allowing to change the deposition direction during the fabrication of the same layer, as well as to work on both sides of the substrate [24]. These characteristics make WAAM technology so attractive to the transportation and civil industry in applications such as structural fabrication.

WAAM technology has attracted the research community’s attention in the last years, and the primary literature includes several experimental studies. However, it is still needed to better understand the process and its adequacy to manufacture parts in different materials meeting the required specifications. Of great interest are the dimensional accuracy and surface quality generated in the process. Proper selection of the welding conditions can help optimize the geometry and surface quality. However, the WAAM process is only adequate as a near-net shape process only as it requires post-processing. In this sense, the stair stepping effect is of great importance due to the large layer thickness that leads to dimensional inaccuracy [16]. Moreover, the capabilities of the process must be evaluated for manufacturing different shapes. Researchers have already studied WAAM for, an example, single bead and vertical walls [25], cylindrical geometries [26] and T-crossing features [27]. However, less attention has been given to the manufacturing of inclined surfaces, which is of great importance [28,29].

The present paper aims to provide readers with an experimental study on the geometry and surface topography obtained when using the WAAM process for manufacturing inclined steel walls. Several manufacturing parameters were studied in a two-stage experimental study.

## 2. Methodology

**Figure 1:**
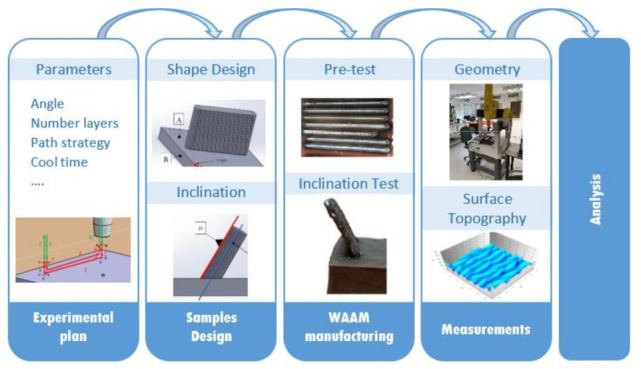
Research methodology.

Figure 1 depicts the methodology used to carry out the study. First, an experimental plan defines the order of the experiments and combinations of the factors (wire speed, travel speed, cooling time, path, layers and angle of inclination) to evaluate the influence of the selected manufacturing parameters, including initial pre-tests, experiments A, B, C, D, E and F. For instance, specifically, the F experiment includes the inclined walls. Second, a design process of shapes and layers helps identify the geometries to manufacture to program trajectories and process conditions. Third, the WAAM process takes place according to the experimental plan. Fourth, the established measuring strategy helped study the samples’ geometry and surface topography. Finally, the obtained results are analyzed.

### 2.1. Materials and Equipment

A solid steel welding wire of 1.2 mm in diameter was selected as the additive material in the welding system (commercial name: AWS ER70S-6). S235JR steel plates were used as substrate. According to ISO 14175 [30], a mixture composed of CO_2_ (15%) and argon (85%) was used as protecting gas. The results of using the mixture gas are stability in the process, improvement in the surface finish quality and reduction of the splatters. The less CO_2_, the smaller the welding drops [31].

The experimental equipment used to carry out the WAAM process is composed of the integration of two different systems:

1. The welding system was a Fronius^®^ TransSteel 3500 with a microprocessor-controlled inverter system with a synergic function suitable for gas metal arc welding. The torch used in this case was a Fronius^®^ MTW5000 model, and the wire feeder was a Fronius VR 5000 Rob 4R/FSC Kenv with double motorized rollers [32]. Depending on the gas used, it works as a MAG (Metal Active Gas) welding process. The welding system has five programable preset jobs. Two jobs were used in this research. Firstly, the wire material and thickness of the substrate must be selected. Then, under the synchronized welding procedure, wire feed, current intensity and voltage must be carefully chosen. The other two parameters will be selected automatically.
2. The positioning system was a six-degrees-of-freedom KUKA^®^ Kr16 robot with anthropomorphic morphology and a nominal load of 16 kg. The operating range was 1611 mm from the center of its base. The wire feeding system was supported on the third axis. The positioning repeatability was 0.05 mm.

**Figure 2:**
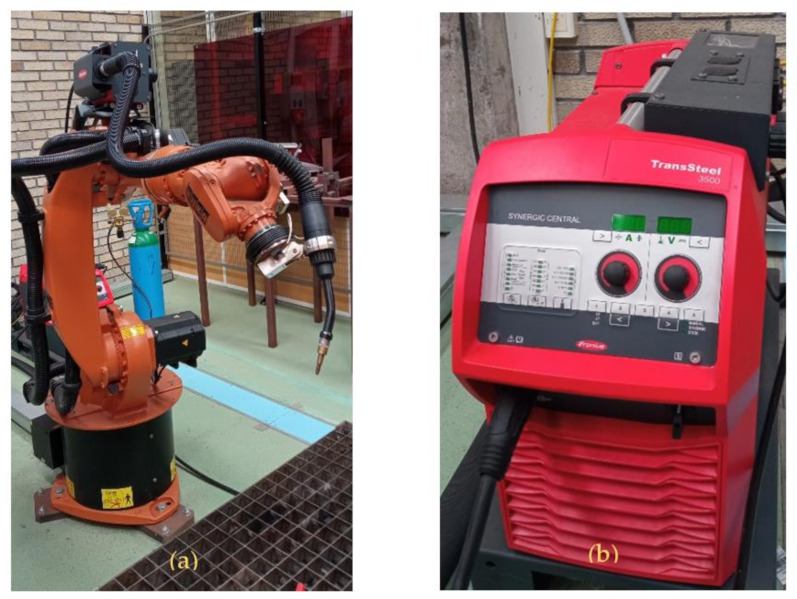
(**a**) Robot KUKA Kr 16, Torch and wire feed motor, (**b**) Fronius power generator Transteel 3500.

The integration of the two systems was made by a Profibus controller. Figure 2 shows the experimental equipment described in this section.

The design of the parts, programming of the robot paths, and post-processing was done using the KRL code with the Delmia^®^ software (Dassault Systemes^®^). The modification of the KRL programs to include the start and stop welding sentences was done with OrangeEdit from OrangeApps (Augsburg, Germany).

### 2.2. Experimental Plan

Process parameters have an important effect on the results of the bead. Among them, the selection of the wire feed rate, travel speed, arc current and heat input can provide different cross-sections of the beads because of width and height variations [6].

The heat input (Equation (1)) is a critical factor that may affect the size and quality of the welds, particularly when depositing material onto previously deposited layers because of the remelting. For instance, in the case of TIG-based WAAM, excessive heat input deteriorates the microstructure, bead geometry and mechanical properties [6,33]. This parameter depends on the travel speed (*S*), average arc current (*I*), arc voltage (*V*), and process efficiency (*η*). The process efficiency ranges from 0.8 to 0.9 [34,35] and, in the present study, is assumed to be 0.8 [36].

(1) $$HI\;\left(\mathrm{kJ}/\mathrm{mm}\right)=\frac{\eta *V\;\left(V\right)*I\left(A\right)}{1000\;\left(J/\mathrm{kJ}\right)*S\;\left(\mathrm{mm}/s\right)}$$

The experimental plan includes two stages. Firstly, a pre-testing stage helped in defining adequate welding conditions to use in the following tests. The WAAM process was used to create seven weld beads on a substrate. Based on the observations, three additional confirmatory tests were tried with adequate, as observed, welding conditions. To perform these tests, the variable parameters selected were the intensity (in synchronous mode), voltage and wire feed, and the travel speed of the robot. Specifically, the factors and levels were intensity (60 and 86 A), voltage (15.4 and 16.4 V), wire feed (65 and 85 mm/min) and travel speed (2, 4, 9 and 20 mm/s). Table 1 lists the combinations of factors and the testing order. The job1 configuration of the welding process corresponds to the conditions of intensity equal to 60 A, the voltage of 15.4 V, and wire feed equal to 65 mm/min. The job2 configuration corresponds to an intensity of 86 A, voltage 16.4 V and wire feed equal to 85 mm/min.

**Figure 3:**
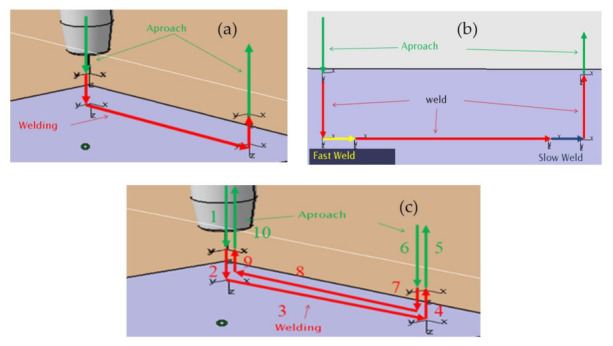
Programming path strategies in Delmia^®^: (**a**) go, (**b**) go with modified enter and exit conditions, (**c**) back (1-2-3-4-5) and forth (6,7,8, 9, 10) path strategy.

The second stage includes six specific tests (A to F) in which several strategies were defined. Travel speeds ranged from 2 to 9 mm/s, and the inclination of the walls from 0 to 90°. Table 2 lists all the details. This set of tests allows for studying the path strategy. Particularly, three path strategies were used: go strategy (test A), go strategy with modified enter (fast weld for the first 5 mm) and exit conditions (slow weld for the last 5 mm) (test B) and back and forth strategy (test C and rest of the tests), as shown in Figure 3.

Lehmann and his team [37] studied the effect of the main welding parameters and the benefits of interlayer intercooling, even using passive cooling methods. The authors identified the influence of varying the travel speed of the robot and wire feed speeds, voltage and arc length on the material deposition and the amount of heat input. The travel speed was particularly decisive for the occurrence of surface rippling. The study by Wang et al. [38] also identified the effectiveness of waiting times and strategy types by comparing continuous zigzag deposition against unidirectional deposition with 24 and 120 s waiting times. Based on these results, the D and E tests included both intermediate cooling and no intermediate cooling to analyze this strategy.

The inclination of the walls was 90° and changed from 60° to 0° in the F tests. Intermediate cooling in these tests was 60 s, being the objective to obtain walls with a length of 70 mm and a height of 50 mm. The analysis of effect of gravity on the material is evaluated by means of the 3D surface of the top and bottom of the wall. In addition, the number of beads and their spacing are corrected in the programming according to the inclination.

### 2.3. Measuring Procedure

**Figure 4:**
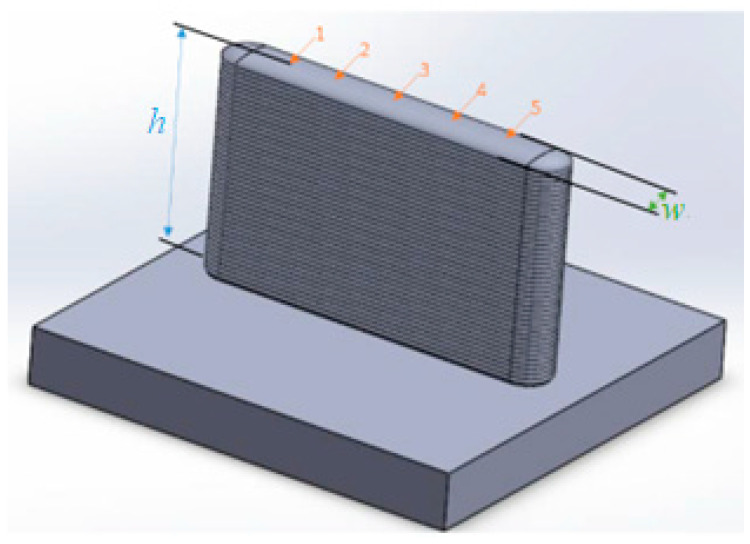
Measuring procedure of the layer’s height (*h*) and width (*w*) at points 1–5.

The measurements in the A, B and C tests were made with a caliper and a depth caliper at five points of each manufactured layer, measuring the height (*h*) and the width (*w*), as shown in Figure 4. The average and standard deviation of these five values were calculated. The standard deviation informs the dispersion of the results and represents an indicator of the process stability. The same measurement procedure was applied to the D test, but only the last layer was measured due to the short cooling time that does not allow measuring layers.

**Figure 5:**
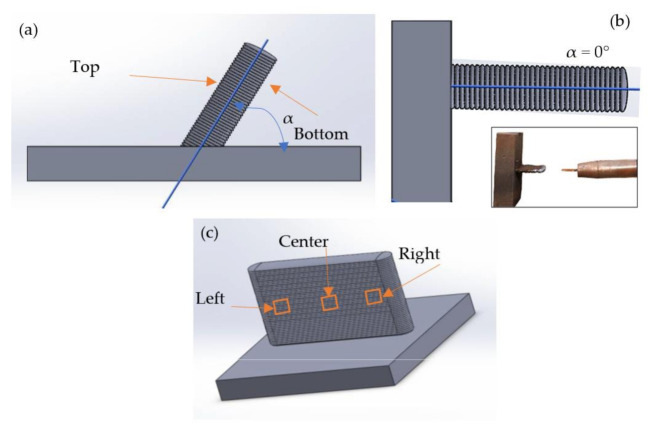
(**a**) Samples E (α = 90°) and samples F1, F2, and F3, (**b**) sample F4, (**c**) position of 3D surface measurements.

The E and F tests were measured with the Trimek Zubi CNC 50.40.30. coordinate measuring machine (CMM) with a TP2 Renishaw probe and 4 mm spherical stylus. The CMM helped to measure the orientation of the walls (α), as shown in Figure 5a,b. Figure 5a shows a generic inclined wall, and Figure 5b shows a horizontal wall in which the substrate lays in the vertical position. Of special interest is the comparison of the top and bottom results because of the possible influence of the heat accumulated in the bottom of the inclined walls.

The 3D surface topography of the inclined walls of E5 (vertical wall, *α_n_* = 90°) and F4 (horizontal wall, *α_n_* = 0°) was measured using an Infinite Focus Alicona^®^ focus variation microscope. The profiles obtained were then processed with MountainsMap^®^ software from Digital Surf^®^ [39] to normalize the surfaces. Table 3 shows the selected parameters, *Sa*, *Sz*, *Ssk* and *Sku*, along with their descriptions and equations, according to the ISO 25178-2 standard [40]. The measurements were taken on each side of the wall (top and bottom) located in three zones of the wall (left, center and right zone), as shown in Figure 5c.

## 3. Results and Discussion

The travel speed of the robot notably influences the cross-section of the beads. As the travel speed decreases, the deposited volume increases, which means that both the width and height of the bead increase.

**Figure 6:**
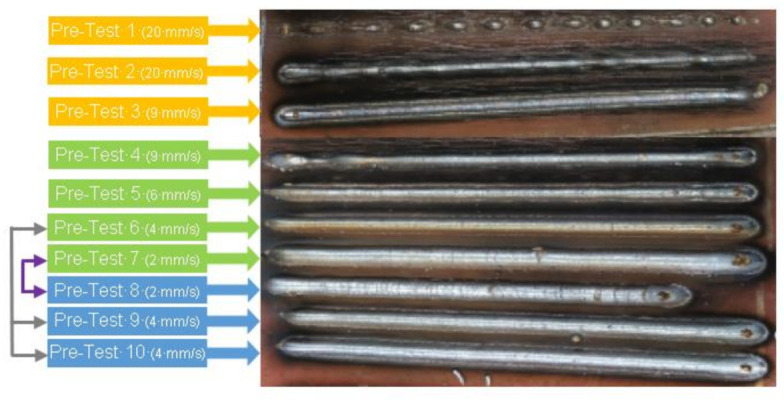
Selection of the optimum beads from pre-tests and confirmatory tests (8–10).

The pre-test results help in defining the best WAAM conditions to use to build steel walls. The conditions used for the three first welds were not adequate because of the poor continuity of the material deposited onto the substrate. Specifically, the travel speed of 20 mm/s proved to be excessive for the welding process. The best conditions were those of the 4, 5, 6 and 7 pre-tests, highlighted in Figure 6. To guarantee the suitability of these conditions, additional tests 8 (same parameters as test 7), and 9 and 10 (same parameters as test 6) were performed. These additional welds showed a similar appearance to the previous ones and, thus, allowed for selecting the conditions for the second stage of the experiment.

Table 4 shows the results of the measurements of the manufactured walls, experiments A, B, C, D, E and F. The table lists the measured values of height and width, along with their standard deviations for height (*SDh*) and width (*SDw*). It also shows the measured angle (*α*) of the inclined wall. Moreover, a quality inspection helped group the tests as those with good and poor quality walls. Good quality was defined as the process resulting in a regular and homogeneous welding wall.

### 3.1. Microstructural Analysis

Microstructure examination of metallographic specimens of previous samples with the same wire material EW70S and material of counterpart was S235JR. The samples used to make this study were made under similar conditions. The same equipment was used with 4 mm/s travel speed. The cool time used between layers was 60 s, and the number of layers was 6, and thus is similar to sample C2.

The analysis of the surface was carried out using an optical Olympus GX-51 Inverted Metallurgical Microscope and the Olympus analySIS FIVE was used as image processor software. Metallographic specimens were prepared by polishing, following etching with Nital solution (5 mL HNO3 and 95 mL ethanol).

**Figure 7:**
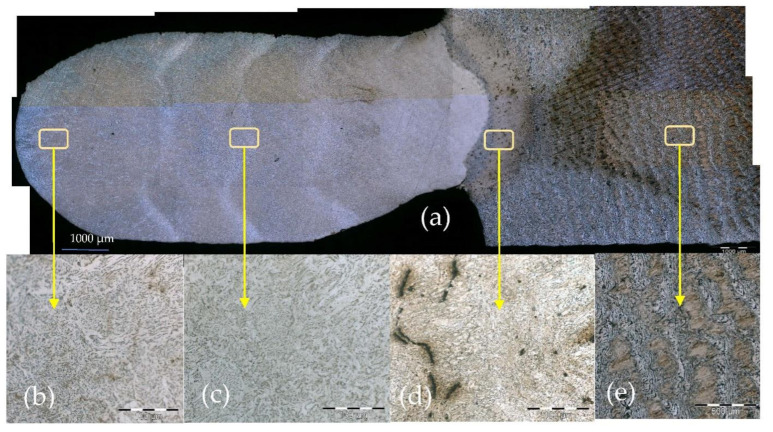
(**a**) Microstructure of printed (WAAM) sample alloy ER70S-6/S235JR obtained by optical microscopy; (**b**) microstructure of the sixth layer; (**c**) microstructure of interstitial 3rd–4th layer; (**d**) porous nd lack of fusion defects on the HAZ zone; (**e**) microstructure of substrate.

Figure 7 shows the microstructure of the alloy section (ER70S-6/S235JR) in the XZ plane produced by the WAAM process. This reveals a typical low-carbon steel microstructure with a ferrite matrix and a relatively small amount of a secondary pearlite phase, as expected from the limited amount of carbon (0.07% C) and the fast solidification conditions of the WAAM process. The microstructure of the reference alloy ST235JR had a regular ferrite matrix and a secondary pearlite phase, as shown in Figure 7e, indicating that this alloy has a very similar microstructure with a relatively larger amount of secondary pearlite phase due to the higher carbon content (0.17% C) present in this alloy. In addition, microstructural imperfections were present as can be distinguished in terms of porosity, impurities and lack of fusion [41,42].

### 3.2. Geometrical Analysis

#### 3.2.1. Travel Speed

Travel speed is a critical factor for the shape of the weld. Related to the results of the pre-tests, it seems that the use of a travel speed of 9 mm/s (test A1) was still too high, and, in this sense, the quality of this bead was poor.

**Figure 8:**
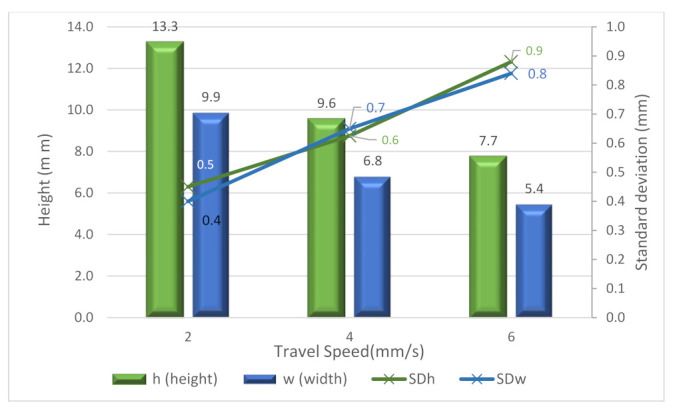
Average height, width and standard deviation (*SDh* and *SDw*) of samples A, B and C.

For the rest of the tests, the cross-section depends largely on the travel speed, as shown in Figure 8. For the A, B and C, tests, it is clear that when the travel speed is lower, both the height and width of the weld increase. This result relates to the heat input. Therefore, as the heat input increases, the size of the bead also increases. The height and width results are mainly the same when the travel speed is constant (see B1.1, B1.2 and B3 tests). Thus, travel speed is a key factor for optimizing the process in terms of productivity. Furthermore, it is possible to see the standard deviations for height (*SDh*) and width (*SDw*). In general, higher deviations appeared for the higher travel speed (6 mm/s).

#### 3.2.2. Path Strategy

**Figure 9:**
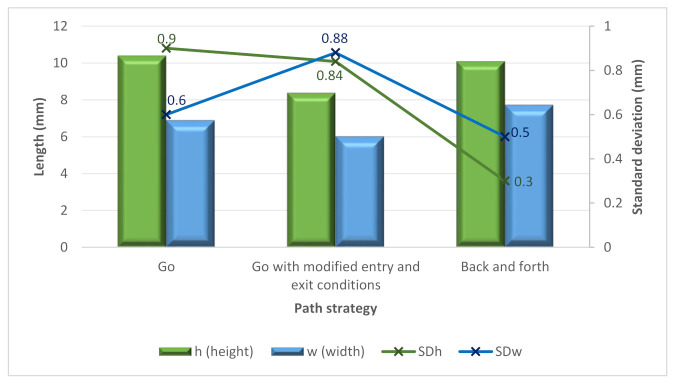
Average height, width and average standard deviations (on samples A, B and C) versus the path strategy.

The B tests were performed by modifying the entering and exiting conditions used in the A tests. Thus, they were performed by increasing the travel speed at the beginning and decreasing the travel speed at the end. This strategy’s effect was a decrease in the height and width of the bead (see Figure 9). Moreover, the “Go” and “Back and Forth” strategies were studied. Regarding the cross-section, no significant differences appeared depending on these two strategies, but some can be noticed when analyzing the variability of the height and width results. In this sense, in Figure 8, it is possible to see how the back-and-forth path strategy provided a decrease in *SDh* and *SDw*. This results in more stable wall conditions.

#### 3.2.3. Intermediate Cooling

**Figure 10:**
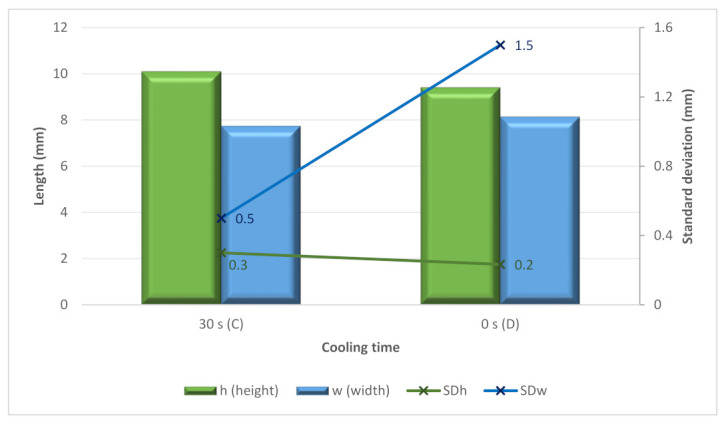
Height, width, and standard deviation for the C and D samples versus the cooling time.

The use of intermediate cooling was analyzed in the C and D tests, comparing the use of no cooling and 30 s of intermediate cooling. The use of no cooling time produced more irregular beads considering the high standard deviation for the width, as shown in Figure 10. It can be noticed that with the cooling time, the *SDw* is significantly lower than without cooling time. Moreover, with six layers, it is possible to observe that the average height with cooling time is higher at 7.4%.

In the next figure, we see a metallographic image of previous experiments of WAMM with six layers.

**Figure 11:**
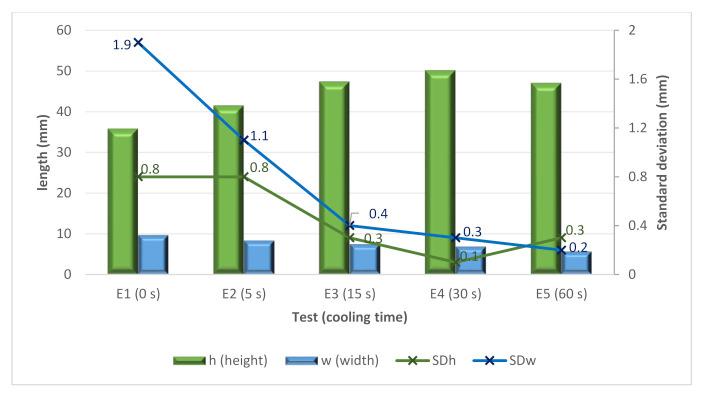
Height, width and standard deviation versus the E samples.

The E tests helped to analyze the influence of the cooling time in more detail. In this sense, cooling times from 0 to 60 s were attempted. These tests were used to create larger walls (up to 45 layers). Figure 11 shows both the height and width and the standard deviations. From the figure, it is possible to see again how higher cooling times improved the stability of the bead. Moreover, there is a positive effect of the cooling time for increasing the height of the bead to a certain point. In contrast, the width moderately decreases when increasing the cooling time.

The results of the surface quality of the walls showed irregularities due to the spattering of the molten metal unable to settle before the material was added at the top level. Thus, the continuous deposition strategy with zero cooling time proved not a suitable strategy.

Based on the results, it was concluded that the cooling time of 60 s, although it considerably increases the total process time, was the most suitable for the E test, as it presents a great gain in height in the direction of deposition and great regularity in width.

#### 3.2.4. Inclination Angle

**Figure 12:**
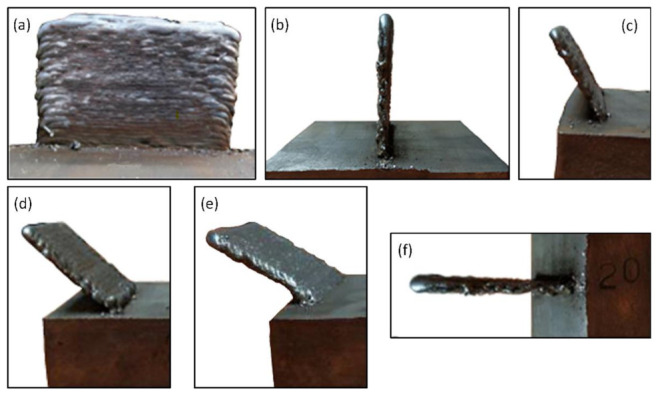
(**a**) Sample E5 *α_n_* = 90°, front view; (**b**) Sample E5 *α_n_* = 90° lateral view; (**c**) Sample F1 *α_n_* = 60°; (**d**) Sample F2, *α_n_* = 45°; (**e**) Sample F3, *α_n_* = 30°; (**f**) Sample F4 *α_n_* = 0°.

In the F test, the aim was to obtain walls with a length of 70 mm and a height of 50 mm, with inclinations *α_n_* of 60°, 45°, 30° and 0° and under the same conditions as the sample E5 (vertical wall, *α_n_* = 90°). It is important to consider the effect of gravity on the material. In addition, the number of beads and their spacing were corrected in the programming according to the inclination. Figure 12 shows the vertical E5 and inclined walls with different inclination angles.

**Figure 13:**
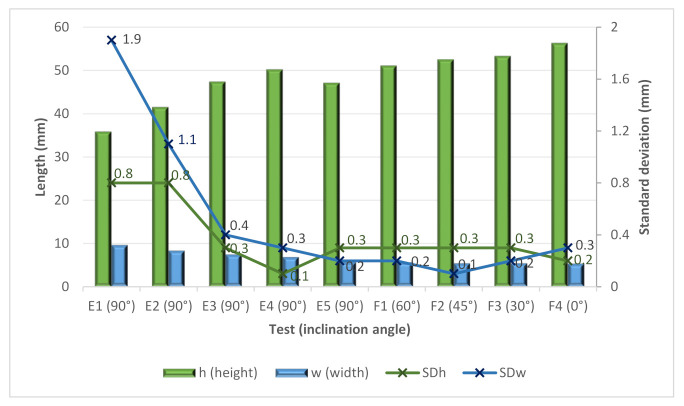
Height, width and standard deviations for the E and F samples.

Figure 13 shows the values for height and width along with the standard deviations. The gravity causes relevant variations in width and height, decreasing and increasing, respectively. The increase in the size of the bead weld must agree with the one programmed, considering that it decreases as smaller angles are used. It is interesting to note how the E3, E4 and E5 tests, which differ in cooling time, provided lower standard deviations. In this sense, it seems clear that no cooling time or a short cooling time (5 s) seems not to be an adequate strategy for obtaining good stability in the welding process, as discussed above. In the case of the F tests, again, a low variation can be seen. Thus, the use of cooling time is also required for obtaining stable inclined walls.

**Figure 14:**
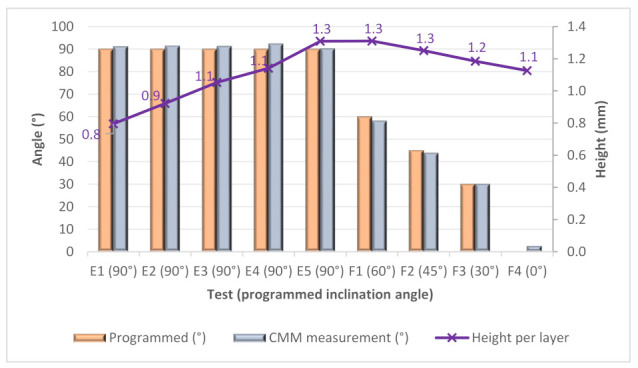
Measure angle, programmed angle and height per layer versus samples E and F.

The manufacturing of complex geometries with high dimensional accuracy is of great importance for the WAMM process to limit the need to perform post-processing by machining. In this respect, to calculate the wall angle tolerances, a coordinate measuring machine measured the angle of inclination, and the results were compared with the programmed path angle. Figure 14 shows the results for E and F tests. The graph shows how close the results are to the programmed results. The maximum tilt error was 2.5° for sample F4 due to the difference between the programmed angle (*α_n_* = 0) and the measured angle (*α* = 2.49°). On average, the error was 1.4° for all nine samples.

In addition, the height per layer was calculated by dividing the total height by the number of actual layers. Figure 14 shows that the height per layer growth obtained is slightly higher than programmed (1 mm/layer), with average increments of 0.25 mm, 0.18 mm and 0.13 mm, in samples F2, F3 and F4, respectively.

### 3.3. Surface Quality

The control of the surface quality is of great importance in engineering. In many mechanical engineering applications, this issue plays a primary role [43,44]. In additive manufacturing, surface topography is considered an important feature of functional character [45,46]. The possibilities of mapping the surface by means of various techniques are analyzed, in addition to those originating from the geometry measurements in the macro scale [47]. Nevertheless, the micro-scale allows for the evaluation of the machining quantitatively by using parameters and functions [48,49]. Thus, the surface topography was evaluated using 3D surface roughness parameters. The topography measurements were made for the E5 and F4 samples that correspond to the vertical and horizontal positions, respectively. Table 5 shows the values of parameters according to ISO 25178, measured at both the bottom and top sides and at different zones of the wall for the sample. The same procedure was taken for the E5 sample on both sides of the sample.

As an early conclusion, it can be highlighted that the surface roughness values are notably high, with the *Sa* always being higher than 20 μm. This suggests that it is likely that post-processing by machining should be performed to guarantee adequate surface quality. Moreover, it can be observed that for the vertical walls, as expected, the measuring side has no influence on the results. However, the measuring side had a notable influence on the results obtained when the inclination angle was 0° as the value of *Sa* 10% was higher on the bottom than on the top. In general, poorer surface quality was observed in the horizontal specimen than in the vertical specimen.

**Figure 15:**
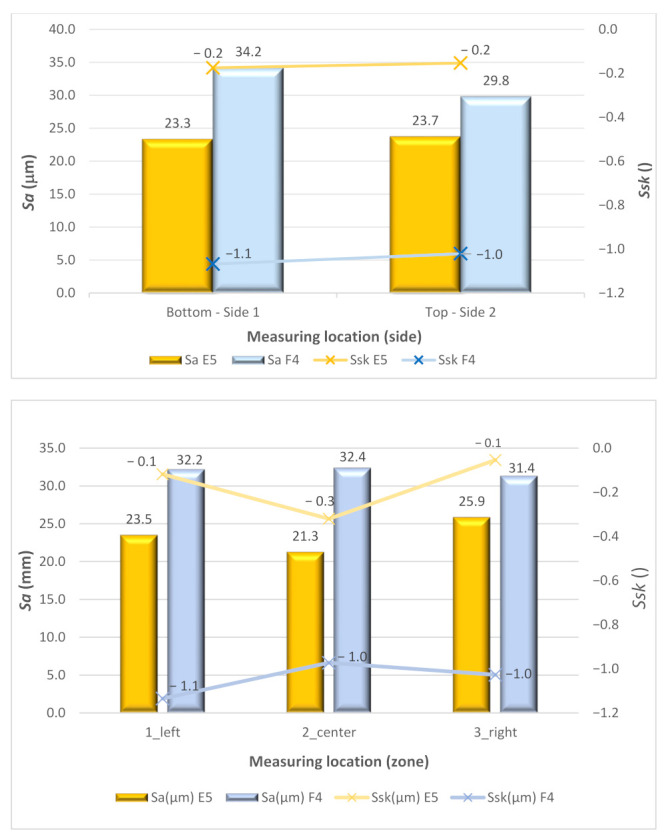
Comparative of 3D surface parameters *Sa* and *Ssk* of samples E5 and F4, versus Measuring location.

Figure 15 shows the behavior of the surface parameters *Sa* and *Ssk* according to ISO 25178 in relation to the measured side. *Sa* increased on the F4 specimen, particularly in the bottom.

**Figure 16:**
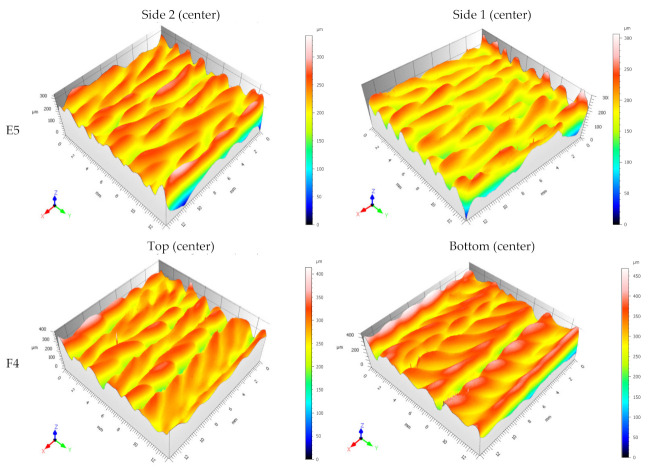
3D topography of the two sides of vertical sample E5 and horizontal sample F4.

Figure 16 shows the 3D topography of the two sides in the center of samples E5 and F4. The selected scale was 300 µm and 400/450 µm for the E5 and F4 cases, respectively. There are no significant differences between the morphology of the topography in the case of E5. The case of sample F4 is different due to the morphology of the surface looking irregular at the bottom. The skewness *Ssk* < 0 indicates the predominance of valley structures in the F4 sample. Moreover, the value of the kurtosis *Sku* gives an indication of the sharpness of the profile. In this case, for the F4 sample, the values are above 3, so it is concluded that the height distribution is spiked.

## 4. Conclusions and Future Work

The present study shows an experimental investigation on the use of the WAAM technology to manufacture inclined walls made of AWS A5.18./ER70s-6 steel. The objective was to analyze the influence of several manufacturing conditions on the geometry and surface topography of the manufactured walls. The following points summarize the main conclusions of the study:

1. The use of simulation robotic control software for the process provided satisfactory results in the manufactured parts, obtaining values similar to the theoretical ones. The software makes it possible to design and readjust the different parameters present in the process quickly and easily.
2. The electrical parameters (voltage and current intensity) and the travel speed are of great importance in the morphology of the parts because they are significant factors responsible for the heat input in each zone and the distribution of the material.
3. The accumulation of heat in the parts is the leading cause of deformations, producing lower height gains and greater width than estimated. Improving heat dissipation will improve the final shape of the manufactured parts.
4. Intermediate cooling in welding significantly improves the results obtained, providing structures with more uniform dimensions, smaller widths and higher layer growth.
5. The inclination of the pieces of walls significantly affected the growth obtained for each bead and its width and uniformity, generating minor imperfections at the bottom of the pieces because of gravity.
6. The use of the WAAM method makes it possible to manufacture inclined structures effectively without the need to move the base parts or use additional supports, making it especially useful for medium and large parts with complex shapes.

Finally, the presented experimental study allows the identification of the following future lines in WAAM technology:

- Influence of gravity on inclined structures of different materials with different densities, such as aluminum, brass and titanium. The authors are currently preparing specimens with different geometries and other materials such as 5356 aluminum. Experiments are also being prepared with specimens with 2319 aluminum in an installation consisting of a Kuka kr16 robot and a Fronius welding system.
- Influence of the inclination on the microstructure of the parts, hardness, residual stresses, and other mechanical characteristics. WAAM should be carefully researched in terms of microstructure and the porosity of the manufactured pieces. This is especially critical when tight mechanical requirements must be met. Work is currently underway on mechanical characterization by creating tensile test specimens and comparing the number of porous obtained by computer tomography. Significant samples will be submitted for metallographic testing to study the microstructure.
- Forced cooling strategies using refrigerated bases, controlled atmospheres or gas jets, which could shorten the processing time and partially control the distribution of the molten material. Future work will take into account the thermographic measurements made with a FLIR thermal imaging camera. The cooling systems are currently under study.

## Figures and Tables

**Table 1:** Experimental plan of pre-tests.

Sample	Weld Process	Intensity (A)	Voltage (V)	Wire Feed (mm/min)	Travel Speed (mm/s)	Heat Input (kJ/mm)
1	Job1	60	15.4	65	20	0.037
2 ^1^	Job1	60	15.4	65	20	0.037
3	Job1	60	15.4	65	9	0.082
4	Job2	86	16.4	85	9	0.125
5	Job2	86	16.4	85	6	0.188
6	Job2	86	16.4	85	4	0.282
7	Job2	86	16.4	85	2	0.564

^1^ All the samples were made with one layer, except sample 2 which was made with two layers to solve errors.

**Table 2:** Experimental plan of the A–F tests.

Test	Travel Speed (mm/s)	Cooling Time (s)	*α_n_* (°)	Path Mode	Layers
A1	9	30	90	Go	5
A2	6	30	90	Go	5
A3	4	30	90	Go	5
A4	2	30	90	Go	5
B1.1	6	30	90	Modified go	5
B1.2	6	30	90	Modified go	5
B1.3	6	30	90	Modified go	5
B2.1	4	30	90	Modified go	5
B2.2	4	30	90	Modified go	5
C1	6	30	90	Back and forth	6
C2	4	30	90	Back and forth	6
C3	2	30	90	Back and forth	6
D1	6	0	90	Back and forth	6
D2	4	0	90	Back and forth	6
D3	2	0	90	Back and forth	6
E1	6	0	90	Back and forth	45
E2	6	5	90	Back and forth	45
E3	6	15	90	Back and forth	45
E4	6	30	90	Back and forth	44
E5	6	60	90	Back and forth	36
F1	6	60	60	Back and forth	39
F2	6	60	45	Back and forth	42
F3	6	60	30	Back and forth	45
F4	6	60	0	Back and forth	50

**Table 3:** Parameters of 3D surface topography according to ISO 25178.

	Description	Equation	
*Sa* (µm)	Arithmetic mean surface height	$S_{a}=\frac{1}{m\times n}\overset{m}{\underset{0}{\int }}\overset{n}{\underset{0}{\int }}\left|Z\left(x,y\right)\right|dxdy$	(2)
*Sz* (µm)	Maximum height, between the highest peak and the deepest valley	$S_{z}=Sp-Sv$	(3)
*Ssk*	Surface skewness (dimensionless)	$S_{sk}=\frac{1}{m\times n\times S_{q}^{3}}\overset{m}{\underset{0}{\int }}\overset{n}{\underset{0}{\int }}Z{\left(x,y\right)}^{3}dxdy$	(4)
*Sku*	Surface kurtosis (dimensionless)	$S_{ku}=\frac{1}{m\times n\times S_{q}^{4}}\overset{m}{\underset{0}{\int }}\overset{n}{\underset{0}{\int }}Z{\left(x,y\right)}^{4}dxdy$	(5)

**Table 4:** Results of the A to F, WAAM tests.

Test	Visual Quality Inspection (Good/Poor)	*h* (mm)	*SDh* (mm)	*w* (mm)	*SDw* (mm)	*α* (°)
A1	Poor	-	-	-	-	-
A2	Good	8.4	1.1	4.9	0.7	-
A3	Good	9.6	0.9	6.4	0.8	-
A4	Good	13.2	0.7	9.4	0.3	-
B1.1	Good	7.7	0.7	5.6	0.5	-
B1.2	Good	7.6	0.8	5.5	0.6	-
B1.3	Good	7.7	1.3	5.6	1.7	-
B2.1	Good	9.5	0.8	6.6	0.4	-
B2.2	Good	9.4	0.6	6.8	1.2	-
C1	Good	7.3	0.5	5.6	0.8	-
C2	Good	9.7	0.2	7.3	0.2	-
C3	Good	13.3	0.2	10.3	0.5	-
D1	Good	7.2	0.3	6.3	1.0	-
D2	Good	9.0	0.2	7.3	1.8	-
D3	Good	12.0	0.2	10.8	1.7	-
E1	Good	35.8	0.8	9.6	1.9	91.3
E2	Good	41.5	0.8	8.3	1.1	91.5
E3	Good	47.4	0.3	7.4	0.4	91.4
E4	Good	50.2	0.1	6.8	0.3	92.4
E5	Good	47.1	0.3	5.6	0.2	90.3
F1	Good	51.1	0.3	5.6	0.2	58.1
F2	Good	52.5	0.3	5.4	0.1	43.8
F3	Good	53.3	0.3	5.4	0.2	30.1
F4	Good	56.3	0.2	5.3	0.3	2.49

**Table 5:** Results of topography measurements according to ISO 25178.

Sample	Side	Zone	*Sa* (µm)	*Sz* (µm)	*Ssk*	*Sku*
E5	Side 1	1_left	24.1	429	0.0618	3.96
E5	Side 1	2_center	20.3	306	−0.285	3.2
E5	Side 1	3_right	25.6	330	−0.301	2.84
E5	Side 2	1_left	22.9	224	−0.298	2.73
E5	Side 2	2_center	22.2	338	−0.356	4.2
E5	Side 2	3_right	26.1	534	0.194	4.4
F4	Bottom	1_left	33.6	707	−1.02	6.97
F4	Bottom	2_center	34.6	468	−0.885	6.38
F4	Bottom	3_right	34.3	730	−1.3	14.4
F4	Top	1_left	30.8	1360	−1.25	16.3
F4	Top	2_center	30.2	502	−1.06	9.12
F4	Top	3_right	28.4	414	−0.754	4.95

## Data Availability

Data are contained within the article.
